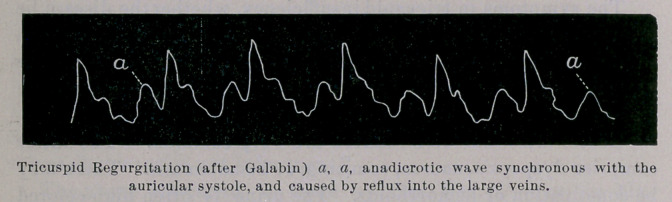# Tricuspid Insufficiency3Read before the Buffalo Academy of Medicine, October 31, 1893.

**Published:** 1893-12

**Authors:** Frank J. Thornbury

**Affiliations:** Demonstrator of Bacteriology, University of Buffalo; 469 Delaware Avenue


### TRICUSPID INSUFFICIENCY.[3]

By FRANK J. THORNBURY, M. D.,
Demonstrator of Bacteriology, University of Buffalo.

"Regurgitation at the tricuspid orifice is generally secondary to mitral stenosis or regurgitation; primary disease of the tricuspid valves, however, is not infrequent."

The above statement is made by Dr. Wm. Pepper, in his admirable System of Medicine, where he devotes no less than three pages to the discussion of this disease. The valvular lesions which lead to tricuspid insufficiency are similar to those which produce mitral insufficiency. The valves are thickened, shrunken, and opaque; the papillary muscles are shortened and thickened. The valves of the cordæ tendineæ and columnæ carneæ may rupture; in either case, acute and extensive insufficiency results. Acute endocarditis of the right heart is rare in adult life, but when it occurs the tricuspid orifices are its primary and principal seat. The first effect of tricuspid regurgitation is dilatation of the right auricle; following this there will be more or less hypertrophy of its walls. As soon as the valves in the subclavian and jugular veins are no longer able to resist the regurgitant current, jugular pulsation follows. The tributaries of the inferior vena cava, and the organs to which they are distributed, become greatly engorged. The liver may present pulsation and, later, assume a nutmeg character in consequence of the continued, chronic congestion. The

1. Krause: *Berlin Klin. Woch.*, 1885, Vol. XXII., p. 462.

2. Rosenberg: *Therap. Monatsheft*, 1888, Nos. 7 and 8. C. H. Knight: *Journal American Medical Association*, 1890, Vol. XIV., p. 89.

3. Read before the Buffalo Academy of Medicine, October 31, 1893.

skin takes on a dingy yellow hue, which, combined with the cyanosis, gives a peculiar greenish tinge that is only met with in heart disease. The condition which I designate *cyanotic induration*, occurring more often under other circumstances, may also be present in this disease. This gives rise to a gastro-intestinal catarrh, or, perhaps, hemorrhoids, which, with ascites, speak for congestion within the abdominal cavity. The spleen becomes enlarged, ordinarily. The kidneys often show cirrhotic changes. Edema of the lower extremities and general anasarca may develop. The obstruction to the systemic circulation may cause hypertrophy of the left ventricle, by an extra amount of work being thrown upon it. Then we have disease of the left ventricle consecutive to that of the right heart.

The symptoms in tricuspid insufficiency, whether the disease be primary or secondary, are for the most part those which pertain to derangements of the abdominal viscera. There may also be present palpitation, cardiac dyspnea, and irregularities in the force and rythm of the heart action.

Gastro-intestinal disturbances are very common. The latter comprise dyspepsia, nausea, vomiting, or hematemesis. There may be constipation or hemorrhoids. The urine is often high-colored and scant, sometimes containing albumin or casts. Cephalalgia, dizziness, and vertigo may be present as indications of cerebral congestion (passive), and there is a peculiar *mental* disturbance, which Pepper regards as *characteristic* of tricuspid insufficiency.

Of especial importance in this disease is the possible disastrous consequence of the assumption of the horizontal posture, as illustrated by the following case. The patient taking the recumbent posture may become cyanosed, and, remaining long recumbent, stupor, coma, and even death may supervene. This fact may be called upon to explain why people are sometimes found dead in bed with heart disease, the case being, perhaps, one of this peculiar type.

According to Dr. Pepper, in no other form of valvular disease is the area of cardiac impulse so markedly increased as in extensive tricuspid insufficiency. This area sometimes extends from the nipple to the xyphoid cartilage, and it may reach as high as the second right intercostal space. Not only the jugular veins pulsate, but also those of the face, arms, hands, and even of the thyroid gland and mamma. The apex beat of the heart is indistinct, and there is commonly epigastric pulsation. Sphygmographic tracings of the pulse show it to be dicrotic.

The area of cardiac dulness, as revealed by percussion, sometimes reaches to the second intercostal space. Auscultation elicits a murmur which is synchronous with, or takes the place, of the first sound of the heart. It is superficial, of low pitch, blowing, soft, and heard best directly over the valves between the fourth and sixth ribs.

The distinctive features of this murmur, as compared with that due to aortic or pulmonary stenosis, or to mitral regurgitation, are, first, its location; second, its character; third, its point of maximum intensity near the base of the ensiform cartilage, and, fourth, the absence of any associated accentuation of the second sound. The presence of jugular and epigastric pulsation are what give weight to the diagnosis in this disease.

Tricuspid Regurgitation (after Galabin) *a*, *a*, anadicrotic wave synchronous with the auricular systole, and caused by reflux into the large veins.

In connection with this presentation of the subject, I desire to report the following case of tricuspid insufficiency, with autopsy:

S. G., male, *æt.* 35 years; single; an American; a farmer by occupation. He gave the following history: had rheumatism four years ago, and was now suffering from "heart and liver disease." There had been progressive weakness for the past six weeks, which, together with shortness of breath and irregular heart's action, necessitated discontinuance of work. There had been edema of the feet and general anasarca, which subsided under treatment. Present condition: patient fairly well developed, of medium height and weight; physique, poor; expression of countenance, haggard; pulse, very feeble and irregular; area of cardiac impulse enormously enlarged, and its outlines imperfectly defined. Patient was intensely dyspneic, and suffering from great mental anxiety. He had walked a long distance prior to coming under observation. He now laid down, and, upon assumption of the horizontal position, immediately died.

Autopsy, fourteen hours after death, revealed the following conditions: body that of an adult male, about thirty-five years of age, well developed; poorly nourished. Rigor mortis is present. Some *post-mortem* staining. Thoracic organs: heart enormously hypertrophied, especially upon its left side. Right auricle and ventricle very much dilated. The tricuspid (right auriculo-ventricular orifice) extremely enlarged, admitting the tips of four fingers, the valves being incompetent. Ante-mortem clots were found in the ventricles of the left side. Liver, intensely congested ; lungs, hyperemic and edematous. Other organs normal.

469 Delaware Avenue.

## Figures and Tables

**Figure f1:**